# Metabolomics of Mice with Type 2 Diabetes and Nonalcoholic Fatty Liver Treated by Acupuncture

**DOI:** 10.1155/2024/5568337

**Published:** 2024-04-10

**Authors:** Yihui Guo, Liying Zhang, Mengyuan Li, Linan Lin, Fuyu Xue, Wanning Gao, Xiaoru Xu, Haipeng Huang

**Affiliations:** ^1^College of Integrated Traditional Chinese and Western Medicine, Changchun University of Chinese Medicine, Changchun 130117, Jilin, China; ^2^Imaging Center, The First Affiliated Clinical Hospital of Guangzhou University of Chinese Medicine, Guangzhou 510405, Guangdong, China; ^3^College of Acupuncture and Massage, Changchun University of Chinese Medicine, Changchun 130117, Jilin, China; ^4^Acupuncture and Massage Treatment Center, The Third Affiliated Clinical Hospital of Changchun University of Chinese Medicine, Changchun 130117, Jilin, China; ^5^Encephalopathy Center, The Third Affiliated Clinical Hospital of Changchun University of Chinese Medicine, Changchun 130117, Jilin, China; ^6^Institute of Acupuncture and Massage, Changchun University of Chinese Medicine, Changchun 130117, Jilin, China

## Abstract

**Introduction:**

To investigate the effects of acupuncture on endogenous metabolites in the liver of type 2 diabetes mellitus (T2DM) with nonalcoholic fatty liver disease (NAFLD) mice-based metabolomics.

**Methods:**

Proton nuclear magnetic resonance (^1^H-NMR) metabolomics combined with multivariate statistical analysis and univariate analysis were used to analyze the changes of endogenous metabolites in the liver of mice in each group and to provide new clinical ideas for acupuncture in the treatment of glycolipid metabolism disorders caused by T2DM and NAFLD.

**Results:**

After 4 weeks of continuous treatment, fasting blood glucose (FBG), insulin (INS), total cholesterol (TC), and triglyceride (TG) decreased significantly in mice in the acupuncture treatment group (ATG), and the content of liver glycogen increased significantly. Based on ^1^H-NMR metabolomic analysis, a total of 47 metabolites were identified in the liver of T2DM with NAFLD mice, of which eight metabolites: UDP-N-acetylglucosamine, adenosine, glutamate, isoleucine, ATP, 3-hydroxybutyric acid, NADP^+^, and leucine were significantly altered by acupuncture treatment. Through the Kyoto Encyclopedia of Genes and Genomes (KEGG) pathway analysis, it is found that acupuncture has an intervention effect on five metabolic pathways, mainly involving amino acid metabolism, energy metabolism, and oxidative stress.

**Conclusion:**

Our study shows that acupuncture can regulate the liver metabolism mode of T2DM in NAFLD mice. It can reduce blood glucose and lipid accumulation in the liver, and these findings provide a new idea and theoretical basis for acupuncture in the treatment of diseases related to glucose and lipid metabolism.

## 1. Introduction

Glucolipid metabolism disorders are characterized by changes in glucose and lipid levels, accompanied by nerve endocrine disorders, insulin resistance (IR), oxidative stress, inflammation, and intestinal flora disturbances as the primary pathological mechanisms, either alone or in combination with dysglycemia, dyslipidemia, high blood pressure, NAFLD, and atherosclerosis as the primary clinical manifestations of the disease. Diabetes mellitus (DM) and fatty liver disease are the most common diseases associated with glucose and lipid metabolism [1]. The global incidence of DM has dramatically increased over the past 40 years, particularly among younger individuals [2]. Notably, DM is approximately three times more likely to die from chronic liver disease than nondiabetic individuals, largely due to NAFLD [3]. Moreover, the incidence of NAFLD increases with elevated blood glucose levels [1]. T2DM and NAFLD interact with each other. Therefore, there is an urgent need to find an effective treatment.

Modern medicine primarily relies on drugs, such as metformin [4], thiazolidinediones [5], silymarin [6], and strictly controlled diets to treat glucose and lipid metabolism disorders. Although these treatments have some effectiveness, they often lead to adverse reactions. Acupuncture, as a traditional treatment method, is based on traditional Chinese medicine (TCM) theory. Studies have shown that acupuncture can significantly improve blood glucose and dyslipidemia levels in patients with glucose and lipid metabolism disorders, promote insulin secretion, reduce cholesterol levels, and regulate gene expression in the liver [7–11]. In addition, acupuncture is widely accepted by patients as a safe and effective treatment method.

Metabolomics is one of the most effective techniques for identifying different metabolites [12]. Its characteristics of high flux, high resolution, and high efficiency make it a convenient tool for clinical and basic research, especially in the field of glucolipid metabolic disorders. Nuclear magnetic resonance (NMR) metabolomics technology, with its advantages of short detection time and small sample size, has been widely used to search for potential disease biomarkers, especially in studying energy metabolism and neurotransmitters [13]. However, ^1^H-NMR spectroscopy is rarely used to investigate the mechanism of acupuncture treatment in T2DM with NAFLD. In this study, we employed a combination of NMR metabolomics and multivariate statistical analysis to analyze the changes of differential metabolites in mice liver, providing a metabolic foundation for the treatment of T2DM with NAFLD by acupuncture and offering new insights for clinical practice.

## 2. Methods

### 2.1. Chemicals

Sodium 3-trimethylsilyl-propionic acid (TSP) was purchased from Qingdao Tenglong Weibo Technology Co., Ltd. (Qingdao, China). Deuterium oxide (D_2_O, 99.9%) was purchased from Shanghai Macklin Biochemical Co., Ltd. (Shanghai, China). HPLC-grade acetonitrile and methanol were purchased from Thermo Fisher Scientific Inc. (Waltham, MA, America). Other chemical reagents were purchased from Thermo Fisher Scientific Inc. (Waltham, MA, America). The liver/muscle glycogen assay kit that was used in this study was purchased from Nanjing Jiancheng Bioengineering Institute (Nanjing, China).

### 2.2. Experimental Animals

All animal experiments were performed at the animal experiment center of the Changchun University of Chinese Medicine. 10 db/m mice (20 ± 2 g, 6 weeks old) and 20 db/db mice (30 ± 2 g, 6 weeks old) were purchased in Changzhou Kavens Experimental Animal Center, License No: SCXK(Su)2016-0010. All mice were raised in SPF level, with temperature 22–24°C, relative humidity 50%–70%, 12 h light alternating day and night, and noise <60 decibels. All mice were fed a highly nutritious diet, with 4–5 mice in each cage. The animals could have free access to food and water. After 1 week of adaptive feeding, 20 db/db mice were randomly divided into the model control group (MCG, *n* = 10) and acupuncture treatment group (ATG, *n* = 10), and 10 db/m mice were used as the normal control group (NCG). This study was approved by the Ethics Committee of the Changchun University of Chinese Medicine (Approval No: 202189). All experiments were performed following the guidelines for the nursing and use of experimental animals of the Changchun University of Chinese Medicine.

### 2.3. Acupuncture Treatment

During the experiment, the ATG mice were given electroacupuncture treatment 1 time a day for 20 minutes, 6 days a week, for 4 weeks in a row. We chose bilateral Feishu (BL13), Pishu (BL20), Shenshu (BL23), Hegu (LI4), Zusanli (ST36), Sanyinjiao (SP6), and Taichong (LR3) as the primary acupoints. Acupoints were located according to the Atlas of Acupuncture Points “Experimental Acupuncture and Moxibustion” (Beijing: China Traditional Chinese Medicine Publishing House Co., LTD, 2016) [14], small mice acuity map for positioning (Table 1). A 10-mm acupuncture needle (Andy) was selected for acupuncturing. The depth of BL13, BL20, and BL23 was 4 mm; ST36 was 3 mm; SP6 was 1.5 mm; and LI4 and LR3 were 1 mm. SDZ-V (Hua Tuo) was selected as the electroacupuncture treatment instrument; BL13 and BL23, SP6, and LR3 on the same side were used as acupoints connected by electroacupuncture; intensive waves were taken with a frequency of 3 Hz (frequency ratio of 1 : 5); and the stimulation intensity was tolerated by the mice. The mice in the MCG and NCG were left untreated and only observed.

After 4 weeks of acupuncture treatment, we collected mice's tail vein blood and injected into a common test tube without anticoagulant, rested, centrifugated, and separated serum. Fasting blood glucose (FBG), INS, triglyceride (TG), and total cholesterol (TC) in serum were determined by a fully automatic biochemical instrument.

### 2.4. Determination of Liver Glycogen Content by Anthrone Method

After 4 weeks of electroacupuncture treatment in each group of mice, inhaled isoflurane anesthetic fully exposed the liver of the mice, and the left leaf of the liver was placed in liquid nitrogen for follow-up testing. After liver sample collection, mice were euthanized by cervical dislocation. Each mouse took 50 mg of liver tissue and added liver tissue and lye to the test tube at 1 : 3 as hydrolysate, boiling water bath for 20 min, flowing water cooling. The liver glycogen hydrolysate was configured into 1% liver glycogen assay solution, and the assay reagents were configured according to Table 2, mixed and boiled in a water bath for 5 min, cooled, and colorimetric at 620 nm wavelength; the OD value of each tube was measured; the standard curve was drawn; the concentration of liver tissue homogenate was calculated from the standard curve; and the liver glycogen content was calculated by the formula.

The formula for calculating liver glycogen content is as follows:

(1) $$\begin{array}{c} \text{Liver glycogen content}\left(\frac{\text{mg}}{g}\text{tissue}\right)=\frac{\text{Measure the OD value of the tube}}{\text{Standard TUBE OD value}}\times \text{standard tube content}\left(0.01\text{mg}\right)\times \text{dilution ratio of the sample before test}\times 10÷1.11. \end{array}$$

### 2.5. ^1^H-NMR-Based Metabolic Profiling

#### 2.5.1. Sample Preparation

After the measurement of basic indicators, 50 mg of liver tissue was weighed before the experiment, homogenized in acetonitrile-water (1 : 1) 1 ml solution, and centrifuged for 10–15 min at 13000 r/min at 4°C. After centrifugation, the solution is divided into two layers, the upper layer is water-soluble material, and the lower layer is fat-soluble material. The supernatant was collected and blow-dried with nitrogen to remove the solvent. The polar extract was dissolved in 700 *μ*L phosphate buffer and centrifuged for 30 seconds, and then centrifuged for 10 minutes at 12000 r/min at 4°C. Before NMR analysis, the supernatant was dissolved in 590 *μ*L NMR buffer (100 mmol/L PBS/D_2_O, PH 7.4, including 0.1 mmol/L TSP), with TSP as the internal standard. The liquid was placed in a nuclear magnetic tube for NMR experiments.

#### 2.5.2. ^1^H-NMR Spectroscopy

^1^H-NMR spectra were collected using Bruker Avance III 600 MHz superconducting high-resolution nuclear magnetic resonance spectroscopy. Cpmgprid standard pulse train was used to record the sample data, and only the presaturation method was used to suppress the water peak. Specific parameters are as follows: standard sampling temperature 298.0 K, parameter spectrum width 12000 Hz, free induction attenuation collection points 64 K, relaxation delay time 2 s, spectrum width 2 × 10^−5^, the cumulative count 256 times, and each scanning time 2.65 s. Detailed phase and baseline adjustments were performed using Bruker Topspin 2.1 software, and the chemical shift of lactate methyl peak (*δ*1.33 ppm) was calibrated.

#### 2.5.3. Data Preprocessing

Use the Mestrelab Research Santiago de Compostela (MestReNova) nuclear magnetic data to import ^1^H-NMR spectrum phase correction and baseline correction. The water peak signal of 4.7∼5.2 was superimposed on all maps, and the region of *δ*0.08∼9.00 was segmented by *δ*0.04. The whole map was normalized by the total peak area. Peak values of ^1^H-NMR per metabolite are obtained using the Human Metabolome Database (HMDB) and from various research papers [15, 16]. Multivariate statistical analysis was performed on all assignments to determine the altered metabolic pattern. The exported data are saved in Text and then imported into an Excel document for data conversion.

#### 2.5.4. Metabolomics

The liver of mice in each group was collected by ^1^H-NMR metabolomics technique and detected by Bruker Avance III 600 MHz superconducting NUCLEAR magnetic resonance spectroscopy. The original NMR data were processed by MestReNova software. SIMCA-p14.0 software was used for differential metabolism statistics of different generations. Metabolic pathway analysis was completed using the MetabaAnlyst 5.0 database and online metabolic data analysis tool, and profile analysis of differential metabolites was performed.

### 2.6. Statistical Analysis

SPSS 22.0 software was used to analyze the data, and the data were expressed as mean ± standard deviation. One-way analysis of variance (ANOVA) was used for comparison between groups, and *P* < 0.05 was considered statistically significant. ^1^H-NMR spectral data were imported into SIMCA-p14.0 (Umetrics, Sweden) for multivariate statistical analysis. First, principal component analysis (PCA) was used to show the grouping trend among all samples. Then, partial least squares discriminant analysis (PLS-DA) was used to compare the MCG and NCG to evaluate the model quality, and 200 permutation tests were performed to verify the PLS model and to judge its validity. S-plot, the variable importance in the projection (VIP), values (>1) to check the differences in metabolite [17]. *R*^2^ and *Q*^2^ were used for the evaluation of PLS-DA. *R*^2^ represents the goodness of fit and prediction ability, and *Q*^2^ > 0.5 represents good model fitting and prediction ability.

### 2.7. Integrated Analysis

To further evaluate the therapeutic effects of acupuncture on mice, the major biological pathways induced by acupuncture were evaluated based on a meta-analysis of significantly altered metabolites, and the relevant metabolic pathways profiles were identified based on MetaboAnalyst 5.0 and the KEGG pathway database.

## 3. Results

### 3.1. Effect of Acupuncture Treatment on Related Indexes of T2DM with NAFLD

**Figure 1:**
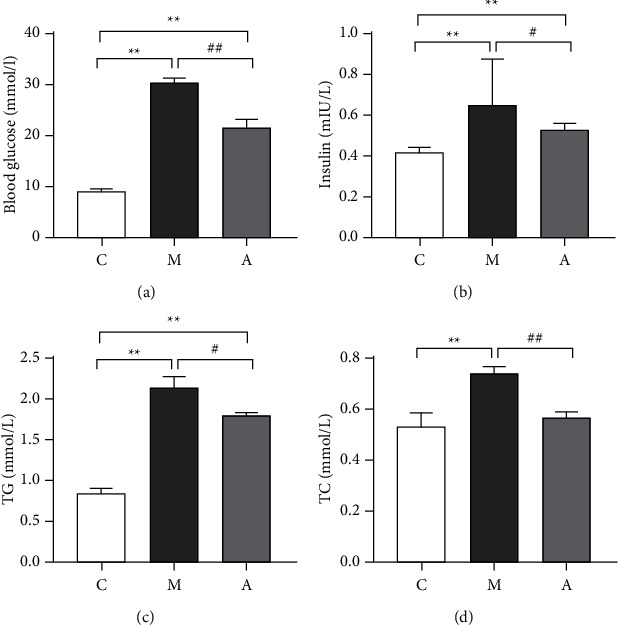
Effect of acupuncture treatment on related indexes of T2DM with NAFLD. *Note*. (a) The changes of FBG in mice of each group after 4 weeks of intervention; (b) the changes of INS level in mice of each group after 4 weeks of intervention; (c) the changes of TG level in mice of each group after 4 weeks of intervention; (d) the changes of TC level in mice of each group after 4 weeks of intervention. Data were evaluated by ANOVA with LSD post hoc tests. ^*∗*^*P* < 0.05, ^*∗∗*^*P* < 0.01 vs NCG group. ^#^*P* < 0.05, ^##^*P* < 0.01 vs MCG group. *N* = 10 mice/group (A = ATG, M = MCG, C = NCG).

The biochemical indexes of mice in each group are shown in Figure 1: compared with the NCG group, the contents of FBG, INS, TG, and TC in the MCG group mice were significantly increased (*P* < 0.01); however, after a 4-week intervention, the value of FBG (*P* < 0.01, Figure 1(a)), INS (*P* < 0.05, Figure 1(b)), TG (*P* < 0.05, Figure 1(c)), and TC (*P* < 0.01, Figure 1(d)) in the ATG group decreased significantly compared to the MCG group. In particular, the TC level tended to be normal after the decrease, and the results were statistically significant. The results showed that acupuncture treatment could improve the basic biochemical indexes of T2DM with NAFLD model mice.

### 3.2. Liver Glycogen Content

**Figure 2:**
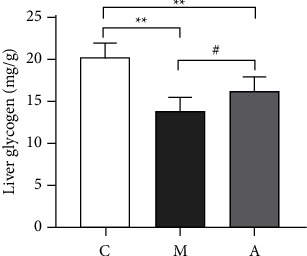
Liver glycogen of T2DM with NAFLD mice. *Note*. ^*∗∗*^significantly different from the NCG (*P* < 0.01), ^#^significantly different from the MCG (*P* < 0.05).

The liver glycogen content of mice in each group is shown in Figure 2: compared with the NCG, the liver glycogen content of mice in the MCG was significantly decreased (*P* < 0.01). Compared with the MCG, liver glycogen content was significantly increased after acupuncture intervention (*P* < 0.05), and the results were statistically significant. These results indicate that acupuncture can significantly reverse the decrease of liver glycogen content caused by T2DM with NAFLD.

### 3.3. Multivariate Analyses of ^1^H-NMR Spectra

**Figure 3:**
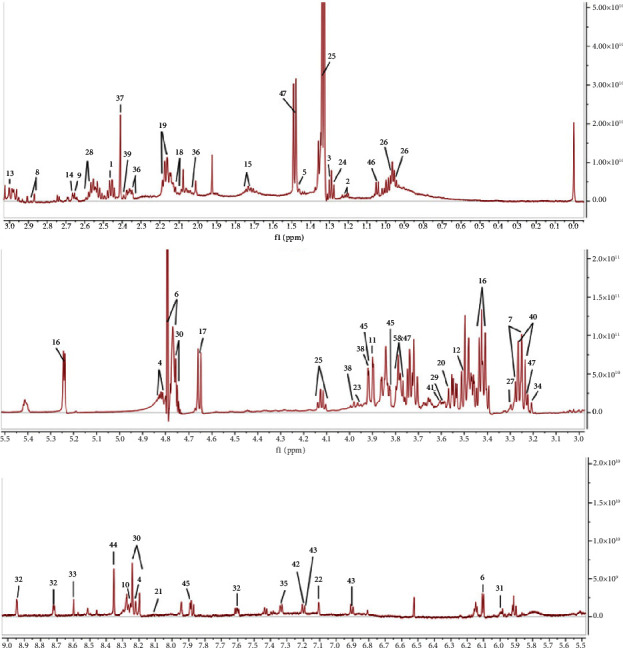
Stack plot of representative ^1^H-NMR spectra from the liver of T2DM with NAFLD mice.

Chemical shifts, peak patterns, and coupling constants were compared using HMDB (https://www.hmdb.ca/) and BMRB (https://www.bmrb.wisc.edu/) databases, combined with relevant research literature [15, 16], which was carried out on the map of small-molecule metabolites related to tag, in the mice liver identified 47 metabolites (Figure 3), including amino acids and organic acids.  Table 3 shows the chemical shifts of all these metabolites.

### 3.4. Multivariate Analysis

**Figure 4:**
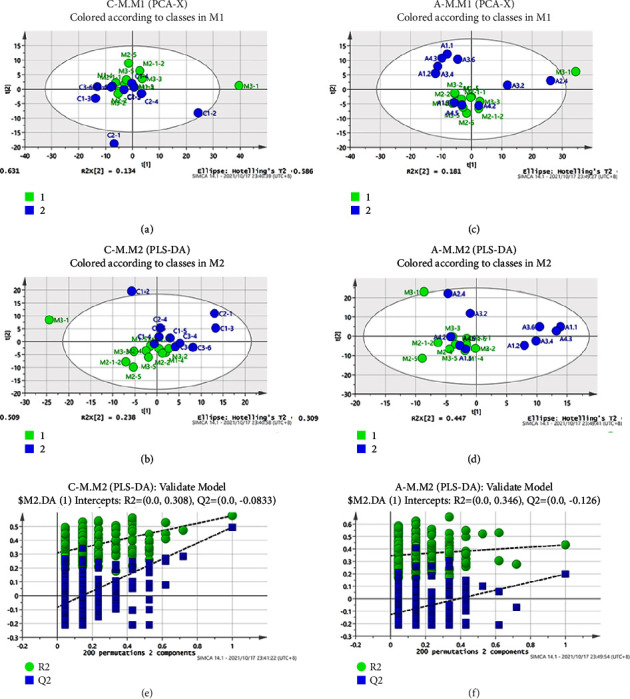
Two-dimensional PCA (a and c) and PLS-DA (b and d) score plots were derived from one-dimensional ^1^H-NMR spectra of the liver from NCG, MCG, and ATG. *Note*. Each data point denotes one subject. A 200 random permutation test for PLS-DA (e and f) models was generated from the liver of NCG, MCG, and ATG. The *R*^2^ value (green) represents the goodness of fit of the model. The *Q*^2^ value (blue) represents the predictability of the models.

To further study the mechanism of “Tiao Zang Tong Luo” electroacupuncture on T2DM mice with NAFLD, based on the information of liver endogenous metabolites obtained by ^1^H-NMR map, PCA and PLS-DA were used to analyze the changes in the overall metabolic profile of mice and determine the differential metabolites. As shown in Figures 4(a) and 4(b), significant group-specific separation was observed in the NCG, MCG, and ATG after PCA. To assess changes in the overall hepatic metabolic profile after acupuncture treatment, we performed supervised PLS-DA clustering based on ^1^H-NMR analysis in a visual form. As shown in the PLS-DA diagram in Figures 4(c) and 4(d), there was a significant separation between the NCG and the MCG (*R*^2^*X* = 0.75, *R*^2^*Y* = 0.58, *Q*^2^ = 0.50), and between the MCG and the ATG (*R*^2^*X* = 0.76, *R*^2^*Y* = 0.43, *Q*^2^ = 0.20), indicating that the metabolic profile changed significantly after 4 weeks of continuous treatment. Based on the VIP value (>1), eight different metabolites (Table 4) were finally identified in the mice's liver according to the metabolic map in Figure 3. To avoid overfitting between the NCG and the MCG, and between the MCG and the ATG, a replacement test was applied (*n* = 200). It can be seen from Figures 4(e) and 4(f) that *R*^2^ and *Q*^2^ arranged on the left are both lower than the original point on the right, indicating that the model of this study is effective and the separation between groups is good.

### 3.5. Integrated Analysis

**Figure 5:**
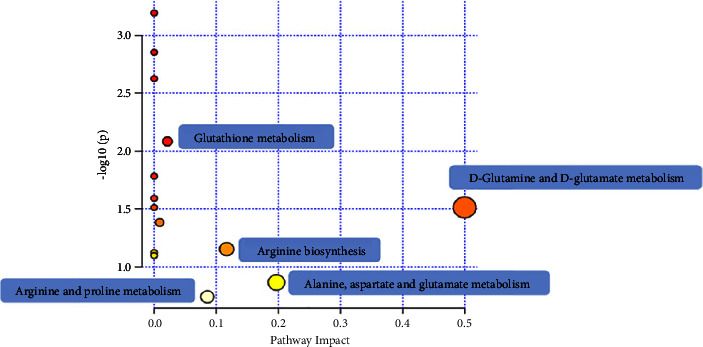
KEGG pathway enrichment analysis.

We analyzed the metabolic pathways of eight integrated differential metabolites. KEGG pathway enrichment analysis of differential metabolites using MetaboAnalyst 5.0 is performed as shown in Figure 5. Compared with the MCG, the influential pathways of the Impact Factor were screened out as potential target pathways, and a total of five metabolic pathways were identified, and it was found that acupuncture may achieve the regulation of glucolipid metabolism disorders by participating in D-glutamine and D-glutamic acid metabolism, alanine, aspartic acid, glutamic acid metabolism, arginine biosynthesis, arginine and proline metabolism, glutathione metabolism, and other metabolic pathways.

## 4. Discussion

The coexistence of T2DM and NAFLD is increasing annually and is causally linked. There is evidence to indicate that T2DM may independently heighten the risk of NAFLD [18], and those suffering from NAFLD are at higher risk of developing T2DM [19]. Blood glucose level serves as a crucial diagnostic marker for diabetes, and INS secretion is regulated by the concentration of blood glucose. Excessive glucose is converted into fatty acids in the liver, resulting in increased fat content within the liver and adipose tissue. The primary feature of early-stage NAFLD is hepatocellular steatosis, characterized by abnormal lipid accumulation in liver cells. Thus, improving blood glucose, blood lipid levels, and hepatic lipid accumulation can effectively mitigate the risk of developing T2DM concomitant with NAFLD [20].

Based on previous research methodologies [21], we established mouse models of T2DM with NAFLD in this experiment to examine the range of pathophysiological mechanisms caused by disturbances in glucose and lipid metabolism. When blood glucose levels increase, it directly triggers pancreatic *β* cells, resulting in a notable increase in INS secretion and subsequently lowering blood glucose levels. In pathological conditions, the functionality of pancreatic *β* cells in the body is compromised. This disruption in insulin secretion results in an inability to facilitate glucose uptake and utilization, leading to the development of insulin resistance (IR). As a compensatory mechanism, the body responds by secreting excessive amounts of insulin to regulate blood glucose levels; however, this compensatory response fails to effectively fulfill its intended role. Our research results indicate that following acupuncture treatment, it has decrease in blood glucose levels and there is a concurrent reduction in INS secretion (Figures 1(a) and 1(b)). Simultaneously, we observed a significant decline in the expression of TG and TC in ATG mice (Figures 1(c) and 1(d)), suggesting that acupuncture effectively mitigates and alleviates hepatic lipid accumulation. Hepatic glycogen is one of the primary sources of blood glucose and serves as an energy storage material formed by the aggregation of numerous glucose molecules in the liver. The synthesis and breakdown of hepatic glycogen are tightly regulated by INS, which plays a critical role in maintaining the stability of glucose levels in the body [22]. Compared to individuals without diabetes, those with diabetes exhibit an accelerated breakdown of hepatic glycogen, leading to increased hepatic glucose output and elevated blood glucose levels [23]. Enhancing the synthesis of hepatic glycogen may serve as an effective strategy for ameliorating abnormal hepatic glucose metabolism in individuals with diabetes. In our research, we observed a significant increase in the hepatic glycogen content (Figure 1) following acupuncture. This finding indicates that acupuncture has the ability to modulate glucose and lipid metabolism in mice, facilitating hepatic glycogen synthesis and exerting a positive influence on reducing blood glucose levels. Consequently, these results suggest that acupuncture, as an adjunctive therapy, holds promise in improving glucose and lipid metabolism while also protecting against hepatic steatosis in diabetic mice.

To elucidate the impact of acupuncture on differential metabolites in the liver of mice with T2DM and NAFLD, we employed ^1^H-NMR metabolomics analysis. Our experimental findings demonstrated the presence of 47 metabolites in the mouse liver, with 8 of them showing altered levels following acupuncture treatment. Subsequently, we conducted a KEGG pathway analysis on these eight differential metabolites. The results revealed that the top 5 enriched pathways in the acupuncture treatment group were all associated with glucose and lipid metabolism, and it indicates that acupuncture regulates the processes of hepatic glucose and lipid metabolism in mice with T2DM and NAFLD.

### 4.1. Acupuncture Can Regulate Amino Acid Metabolism

Under pathological conditions characterized by disrupted glucose metabolism, the process of glucose breakdown is inhibited [24]. As a consequence, there is an elevation in glucose levels within the liver, leading to a decrease in the production of pyruvic acid and lactate. Ultimately, this inhibition affects the glycolysis pathway while concurrently upregulating the gluconeogenesis pathway. Amino acids, as essential components of the human body, play crucial roles in numerous metabolic pathways. Glutamate, isoleucine, leucine, and the metabolic pathways associated with them are all categorized within amino acid metabolism. Glutamate acts as a central hub and intermediary in the metabolism and conversion of various amino acids. Through the regulation of glutamate metabolism, it can have an impact on the metabolic levels of several other amino acids. Glutamate is involved in D-glutamine and D-glutamate metabolism, alanine, aspartate and glutamate metabolism, arginine biosynthesis, and arginine and proline metabolism. It has been identified as a potential biomarker for both T2DM and NAFLD [25, 26], with its alterations in levels being considered highly effective biomarkers among the identified amino acids. Studies have indicated that glutamate levels remain consistently elevated in NAFLD and other metabolic disorders [27], which aligns with the observed increase in glutamate content in the liver tissue of MCG mice. Interestingly, in our study, a significant reduction in glutamate levels was observed in the liver tissue of ATG mice when compared to the MCG group. This suggests that acupuncture may modulate the metabolic function of mice with T2DM and NAFLD by regulating glutamate metabolism.

The degree of IR is closely linked to amino acid metabolism, particularly branched-chain amino acids (BCAAs) [28]. Studies have demonstrated that BCAAs can elevate the mRNA and protein levels of INS in pancreatic *β* cells, thereby playing a role in regulating blood glucose metabolism and maintaining lipid homeostasis [29]. Isoleucine and leucine are essential BCAAs for mammals. BCAAs are closely associated with the risk of developing T2DM. Supplementation with BCAAs has been found to have beneficial effects on various liver diseases [30]. In particular, isoleucine can inhibit hepatic gluconeogenesis, stimulate muscle glucose uptake, and promote overall glucose oxidation, resulting in blood glucose reduction [31]. Leucine enhances the action of INS, leading to increased glucose uptake and utilization in tissues, such as the liver, muscle, and heart. It also improves glucose transport in the liver, significantly reducing glycogen breakdown and gluconeogenesis [32]. Leucine also contributes to improving INS signaling in adipose tissue and reducing inflammation [33]. This downregulates fatty acid synthesis and transport, resulting in a decrease in hepatic lipid accumulation [34].

### 4.2. Acupuncture Can Regulate Energy Metabolism

Adenosine, by acting on A1 receptors, increases intracellular levels of cAMP and calcium ions, thus activating glycogen phosphorylase. In the presence of normal or high glucose levels, adenosine can stimulate insulin secretion in pancreatic islets. The signaling of adenosine is closely associated with glucose homeostasis and the pathophysiology of T2DM, and it also plays a role in regulating adipocyte function. Adenosine receptors are expressed in mouse pancreatic islets and rodent insulinoma cell lines (such as rat INS-1 and mouse *β*TC-6 cells), suggesting the involvement of adenosine in the regulation of *β*-cell function. Adenosine plays a significant role in energy transfer as same as adenosine triphosphate (ATP). It has the ability to reduce tissue inflammation and improve the stability of glucose and lipid levels. Additionally, adenosine can offset the impact of INS on hepatic net glucose output by interfering with the functions of adipose tissue and the liver, as well as regulating immune events related to IR, thus affecting the pathophysiological processes of T2DM. Furthermore, adenosine gradually emerges as a primary regulatory factor in INS response by controlling INS signaling in adipose tissue and the liver. Our research has shown that acupuncture can regulate adenosine levels [35, 36], which is consistent with our findings. We speculate that adenosine receptors may become a potential target for treating T2DM with NAFLD. However, further research is needed to gain a deeper understanding of the adenosine signaling transduction mechanism in the pathophysiology of T2DM with NAFLD. In addition, glucose, fatty acids, and amino acids can be metabolized through various pathways to produce acetyl-CoA, which then enters the tricarboxylic acid cycle for further oxidation and phosphorylation, generating ATP. ATP plays an important role in regulating INS synthesis and secretion in pancreatic *β* cells and liver cells. Dysfunction in *β* cells and IR are the two primary factors leading to T2DM [37–39]. Impaired ATP production and release can result in pancreatic *β*-cell dysfunction. Restoring ATP production and release holds significant potential in rectifying *β*-cell dysfunction and treating diabetes.

UDP-N-Acetylglucosamine is a high-energy compound and one of the substrates required for the synthesis of glycolipids. In the biosynthetic pathway of glycolipids, UDP-N-acetylglucosamine reacts with glycosyltransferases to combine glucosamine molecules with other sugar molecules, forming various types of glycolipids. These glycolipids can be present on the cell membrane and participate in cell recognition, signal transduction, and the composition of the extracellular matrix. Increasing levels of UDP-N-acetylglucosamine have an impact on glucose uptake in response to INS stimulation. Additionally, it serves as a necessary building block for the glycosyl side chains of lipids [40]. Elevated levels of glucose also lead to increased glycosylation of UDP-N-acetylglucosamine, resulting in its significant upregulation in disorders related to glycolipid metabolism [41]. 3-Hydroxybutyric acid can be produced through the ketone body pathway. It is synthesized in the liver and released into the bloodstream, where it can be utilized by other tissues [42]. Ketone bodies are an essential source of energy for extrahepatic tissues. They provide acetyl-CoA and acetoacetyl-CoA for the synthesis of cholesterol, fatty acids, and complex lipids, which may contribute to the increased incidence of NAFLD. In addition, some studies have suggested that 3-hydroxybutyric acid may exert regulatory effects on processes such as fatty acid synthesis and breakdown by influencing cellular signaling pathways.

Compared to the MCG mice, ATG mice exhibited a downregulation of adenosine and ATP levels, as well as a decrease in UDP-N-acetylglucosamine and 3-hydroxybutyric acid levels. These four metabolites are closely involved in energy metabolism, indicating that acupuncture may regulate the imbalance of glucose and lipid metabolism associated with T2DM with NAFLD from an energy metabolism perspective. This has significant therapeutic implications for clinical patients.

### 4.3. Acupuncture Can Regulate Oxidative Stress

Oxidative stress refers to a negative effect caused by excessive production of reactive oxygen species (ROS), which represents an imbalance between oxidation and antioxidant activities within the body. It can result in cellular damage and ultimately lead to functional impairment of cells. NADP^+^ is the oxidized form of the coenzyme II, NADPH (reduced form), and serves as a cofactor involved in various synthetic metabolic reactions. It is utilized for the synthesis of lipids, nucleic acids, and other processes that require NADPH as a reducing agent. NADPH is an essential component of the cellular antioxidant defense system and plays a critical role in protecting cells against oxidative damage caused by ROS. ROS can directly damage pancreatic islet cells and promote cell apoptosis. They can also indirectly inhibit cellular function by affecting INS signaling pathways. When pancreatic islet cells are damaged, it can lead to a decrease in INS secretion levels and delayed peak secretion. This results in exacerbated blood glucose fluctuations and more significant damage to the cells. After acupuncture treatment, there is a significant increase in NADP^+^, which may subsequently be reduced to NADPH. This can help regulate oxidative stress and improve pancreatic islet cell function, thus playing a therapeutic role in correcting abnormal blood glucose and INS levels.

## 5. Conclusion

In conclusion, this study was conducted using mouse models of T2DM with NAFLD. It found that acupuncture effectively alleviates T2DM with NAFLD by regulating metabolites associated with glucose and lipid metabolism, energy metabolism, oxidative stress, and other metabolic pathways. Specifically, the main metabolic pathways affected include D-glutamine and D-glutamate metabolism, alanine, aspartate, and glutamate metabolism; arginine biosynthesis, arginine, and proline metabolism; and glutathione metabolism. Furthermore, by analyzing biochemical markers and hepatic glycogen levels in mice, the study observed that acupuncture has the potential to lower blood glucose levels, reduce lipid accumulation in the liver, and decrease the likelihood of complications. These discoveries offer new insights and establish a theoretical foundation for the application of acupuncture in treating disorders related to glucose and lipid metabolism.

## Figures and Tables

**Table 1:** Specific treatment methods of “Tiao Zang Tong Luo” electroacupuncture.

Acupoints	Positioning	Treatment
Feishu (BL13) (double)	Third thoracic vertebra below both intercostal spaces	0.5-inch needle, puncture obliquely 4 mm
Pishu (BL20) (double)	Twelfth thoracic vertebrae under both intercostal spaces	0.5-inch needle, puncture obliquely 4 mm
Shenshu (BL23) (double)	Second posterior lumbar vertebrae in the two lateral depressions	0.5-inch needle, puncture obliquely 4 mm
Hegu (LI4) (double)	Between the 1st and 2nd metacarpal of the forelimb	0.5-inch needle, puncture perpendicularly 1 mm
Zusanli (ST36) (double)	Below the knee joint, in the muscle groove about 0.3 cm below the fibular tuberosity	0.5-inch needle, puncture perpendicularly 3 mm
Sanyinjiao (SP6) (double)	0.5 cm above the tip of the inner ankle of the hind limb	0.5-inch needle, puncture perpendicularly 1.5 mm
Taichong (LR3) (double)	Posterior recess of the 1st metatarsal space	0.5-inch needle, puncture perpendicularly 1 mm

**Table 2:** Preparation method of liver glycogen detection reagent.

	Blank tube	Standard tube	Measurement tube
Distilled water (ml)	1.0		0.9
0.01 mg/ml standard solution (ml)		1.0	
Glycogen detection solution (ml)			0.1
Coloring solution (ml)	2	2	2

**Table 3:** Assignments of ^1^H-NMR spectra peaks obtained from the liver of mice.

No	Metabolite	*δ* 1H (multiplicity)
1	2-Oxoglutarate	2.45 t
2	3-Hydroxybutyric acid	1.20 d, 1.21 d
3	Acetate	1.32 d
4	Adenosine	4.8 t, 8.2 s
5	Alanine	1.48 d, 3.78 q
6	AMP	4.79 t, 6.12 d
7	Arginine	3.27 t
8	Asparagine	2.85 dd
9	Aspartate	2.66 dd
10	ATP	4.56 t, 8.24 s
11	Betaine	3.89 s
12	Choline	3.50 dd
13	Creatine	3.01 s
14	Citrate	2.68 d
15	DSS	1.74 m
16	*α*-Glucose	3.42 m, 5.23 d
17	*β*-Glucose	4.66 d
18	Glutamate	2.12 m, 2.32 m
19	Glutamine	2.18 m
20	Glycine	3.57 s
21	GTP	8.12 s
22	Histidine	7.11 s
23	IMP	3.94 s
24	Isoleucine	1.25 m
25	Lactate	1.34 d, 4.10 q
26	Leucine	0.94 d. 0.95 d
27	Methanol	3.33 s
28	Methionine	2.63 t
29	Myo-inositol	3.60 t
30	NAD^+^	4.75 t, 8.20 t
31	NADP^+^	6.04 d
32	Niacinamide	7.58 q, 8.70 d, 8.92 d
33	Nicotinate	8.59 d
34	O-Phosphocholine	3.21 s
35	Phenylalanine	7.31 m
36	Proline	2.03 m, 2.33 m
37	Pyruvate	2.41 s
38	Serine	3.93 dd, 3.97 dd
39	Succinate	2.39 s
40	Taurine	3.26 t
41	Threonine	3.61 s
42	Tryptophan	7.19 t
43	Tyrosine	6.91 m, 7.19 m
44	UDP-N-acetylglucosamine	8.35 d
45	Uridine	3.80 dd, 3.90 dd, 7.86 d
46	Valine	1.04 d
47	*β*-Alanine	1.48 t, 3.17 t, 3.78 t

*Note*. s is single-peaked, d is dual-peaked, t is triple-peaked, q is quadruple-peaked, m is multipeak, and dd is double of doublet.

**Table 4:** NCG compared with liver differences in MCG; MCG and ATG mice with metabolite changes.

No	Metabolites	MCG vs NCG			ATG vs MCG		
		VIP	*P* value	Change^a^	VIP	*P* value	Change^b^
1	UDP-N-acetylglucosamine	1.03	0.03	↑	1.04	0.04	↓
2	Adenosine	1.06	0.05	↓	1.03	0.03	↑
3	Glutamate	1.39	0.02	↑	1.54	0.04	↓
4	Isoleucine	1.27	0.02	↓	1.50	0.04	↑
5	ATP	1.19	0.03	↓	1.45	0.01	↑
6	3-Hydroxybutyric acid	1.70	0.05	↑	1.42	0.05	↓
7	NADP^+^	1.13	0.05	↓	1.35	0.04	↑
8	Leucine	1.08	0.06	↓	1.31	0.01	↑

*Note*. The VIP value > 1, a↑ indicates that compared with NCG, the average integral area of the substance at this chemical displacement increases, ↓ is reduced; b↑ indicates that compared with MCG, the average integral area of the material at this chemical displacement increases, ↓ is decreased.

## Data Availability

The data used to support the findings of this study are included within the article. Any further data can be made available from the corresponding author upon request.
